# Interpreting Financial Capacity Research to the Larger Society

**DOI:** 10.1093/geroni/igaf122.1250

**Published:** 2025-12-31

**Authors:** Peter Lichtenberg

**Affiliations:** Wayne State University, Detroit, Michigan, United States

## Abstract

Scholarship and service regarding financial capacity assessment and best practices led to a program of research on financial capacity. This lecture will focus on key elements of that research including financial decision-making, financial exploitation and personal financial behaviors and how each of these link to cognitive functioning and early memory loss in older adults. Our expansion of this work into interventions with older adult victims of scams and identity theft lead us to interpreting our work to older adults and the professionals who work with them. The creation and continual expansion of our website https://olderadultnestegg.com gave us one platform for offering training, intervention and risk assessment to older adults, caregivers and professionals alike. Using a prevention science approach, we created and piloted a prevention of financial exploitation program. This presentation will tie together how leadership principles guide our work.

